# Flexible Piezoresistive Sensor with the Microarray Structure Based on Self-Assembly of Multi-Walled Carbon Nanotubes

**DOI:** 10.3390/s19224985

**Published:** 2019-11-15

**Authors:** Peng Zhang, Yucheng Chen, Yuxia Li, Yun Zhao, Wei Wang, Shuyuan Li, Liangsong Huang

**Affiliations:** 1 Key Laboratory for Robot Intelligent Technology of Shandong Province, Shandong University of Science and Technology, Qingdao 266590, China; sdustzhangpeng@163.com (P.Z.); sdustchenyucheng@163.com (Y.C.); yuxiali2004@sdust.edu.cn (Y.L.); sklishuyuan@163.com (S.L.); 2 Inner Mongolia Aerospace Honggang Machinery Corporation Limited, Huhhot 010076, China; 15560711017@163.com; 3 College of Electronics and Information Engineering, Shandong University of Science and Technology, Qingdao 266590, China; skdwangwei1zqf@163.com

**Keywords:** flexible piezoresistive sensor, flexible strain sensor, multi-walled carbon nanotubes, self-assembly

## Abstract

High-performance flexible pressure sensors have great application prospects in numerous fields, including the robot skin, intelligent prosthetic hands and wearable devices. In the present study, a novel type of flexible piezoresistive sensor is presented. The proposed sensor has remarkable superiorities, including high sensitivity, high repeatability, a simple manufacturing procedure and low initial cost. In this sensor, multi-walled carbon nanotubes were assembled onto a polydimethylsiloxane film with a pyramidal microarray structure through a layer-by-layer self-assembly system. It was found that when the applied external pressure deformed the pyramid microarray structure on the surface of the polydimethylsiloxane film, the resistance of the sensor varied linearly as the pressure changed. Tests that were performed on sensor samples with different self-assembled layers showed that the pressure sensitivity of the sensor could reach $-2.65{\;\;\mathrm{kPa}}^{-1}$, which ensured the high dynamic response ability and the high stability of the sensor. Moreover, it was proven that the sensor could be applied as a strain sensor under the tensile force to reflect the stretching extent or the bending object. Finally, a flexible pressure sensor was installed on five fingers and the back of the middle finger of a glove. The obtained results from grabbing different weights and different shapes of objects showed that the flexible pressure sensor not only reflected the change in the finger tactility during the grasping process, but also reflected the bending degree of fingers, which had a significant practical prospect.

## 1. Introduction

With the development of technology and market requirements, high-performance flexible pressure sensors have been widely used in numerous applications, including robot skin [1,2], intelligent prosthetic hands [3,4], special operation robots [5,6], service robots and wearable devices [7,8]. On the other hand, a sensor should meet the requirements of sensing characteristics such as the sensitivity, response speed and high repeatability. According to the conducted studies, the sensing mechanism of flexible pressure sensors can be roughly divided into capacitive sensors [9,10,11], piezoelectric sensors [12,13,14], transistor sensors [15,16,17] and piezoresistive sensors [18,19,20]. It should be indicated that different types of sensors have different advantages in accordance with their sensing principles. For example, the capacitive sensor is composed of basic capacitor units. Moreover, its basic structure consists of two electrode plates and the sandwiched dielectric. The external stress is reflected through the variation in the capacitance. This sensor has remarkable advantages, including a simple structure, reasonable dynamic response and high sensitivity. Zhenan Bao’s research group [21] applied field effect transistors to capacitive sensor design and integrated a thin film with a microarray structure into an organic field effect transistor (FET) as a dielectric layer, presenting a new type of active sensor device with a reasonable sensitivity and response time. They showed that this method could produce a large area and highly flexible electronic skin so that the excellent sensitivity could detect the ultra-light pressure of a butterfly at stopping. Moreover, Lipomi et al. [22] used a polydimethylsiloxane (PDMS) film with a carbon nanotube coating to produce a transparent and retractable capacitive sensor array for detecting the pressure and strain force. Pruvost et al. [23] designed a high-sensitivity low-voltage detection capacitive sensor based on conductive carbon black particle-filled composite foam with a sensitivity of $35{\;\mathrm{kPa}}^{-1}$($<0.2{\;\mathrm{kPa}}^{}$). Although the capacitive sensor had a high sensitivity and a fast response, it had some drawbacks, including a small detection range, complicated capacitance signal acquisition system, easy signal interference and poor signal stability and repeatability. It should be indicated that these shortcomings originated from the limitation of the capacitive sensor principle. The working principle of the piezoelectric is expressed as the following: when piezoelectric materials are subjected to stress, they deform and generate a voltage to detect pressure, which has the characteristics of a simple structure, high sensitivity and high reliability. Choi et al. [24] applied the polyvinylidene fluoride (PVDF) piezoelectric film and successfully developed a piezoelectric sensor to detect signals of heartbeat and lung breathing during sleep. They utilized a PVDF film and conductive fabric to prepare a belt-type piezoelectric sensor that could be used to detect cardiopulmonary status. However, piezoelectric sensors can only measure the dynamic strain force and they are susceptible to external temperature. Moreover, transistor sensors are characterized by reliable performance and a fast response time. Loi et al. [15] processed the organic field effect transistor (OFET) on the substrate of a flexible PET film through the micro-electromechanical systems (MEMS) fabrication process and applied it to the field of wearable devices to detect human parameters. However, this type of sensor has a complicated preparation process and high cost, which is not beneficial to mass production.

Flexible piezoresistive sensors are the most widely used sensors due to their remarkable advantages, including simple fabrication, convenient signal acquisition, stability and reliability. It should be indicated that the variation rate of the resistance is the key parameter for evaluating the sensor performance. The variation rate of the resistance mainly depends on the magnitude of the internal resistance change of the conductive sensing material or the magnitude of the contact resistance change originating from the deformation of the external structure [25]. It should be indicated that the effective improvement of the variation rate of the resistance, is the existing challenge for improving the performance of flexible resistive sensors. Reviewing the literature indicates that an effective way of improving the variation rate of the resistance of flexible resistive sensors is to select suitable polymer nanomaterials, including carbon nanotubes [26], graphene [27], nanowires [28] and other metal nanomaterials [29]. It should be indicated that the price of graphene and nanowiresis relatively high. Moreover, due to the inherent rigidity of metal nanomaterials, it is a challenge to modify them in a short time. Therefore, multi-walled carbon nanotubes (MWNTs) have recently attracted many researchers because of their moderate price and excellent chemical and physical properties, which can be used to improve the sensor performance. It should be indicated that another way to improve the performance of the piezoresistive sensor is to introduce regular-shaped, irregular-shaped microstructures and foam-like microstructures onto the surface of the piezoresistive material. Regular-shaped microstructures include the semicircular [30], columnar [31], filament [17] and the pyramidal [32]. Moreover, irregular-shaped microstructures include the Gaussian surface [33], mimic epidermal microstructure [34] and the leaf surface [35]. Finally, foam-like microstructures include polyurethane foam [36] and a sugar cube based PDMS foam [37]. It should be indicated that compared with sensors without microstructures, sensors with microstructures can increase the contact area between the sensing material and the electrodes, which can greatly improve the performance of sensors. Recently, many methods have been proposed to improve the performance of piezoresistive sensors. For instance, inspired by the interlocking structure of hair, Pangetal. [31] obtained a high performance piezoresistive sensor by using the surface-modified metal nanowire array to achieve the detection of pressure, shear and torsion. Moreover, Park et al. [26] infused nanosphere arrays using a mixture of carbon nanotubes and PDMS to obtain a high sensitivity piezoresistive sensor with sensitivity up to $-15.1{\;\mathrm{kPa}}^{-1}$(<0.5 kPa). Zhu et al. [25] designed a resistive sensor made of graphene materials combined with PDMS films with pyramid microarray structures, which obtained a sensitivity of up to $-5.53{\;\mathrm{kPa}}^{-1}$(<100 kPa). It should be indicated that the sensitivity of the microstructured sensors was 50 times more than that of the non-microstructured sensors under the same conditions. Park et al. [20] used a heat-shrinkable film and carbon nanotubes to make a wrinkled conductive film. Song et al. [38] proposed a foam PDMS film made of sugar cubes combined with carbon nanotubes as piezoresistive layers. They observed that when an external force was applied to the sensor, the distance between the electrodes reduced and the resistance correspondingly increased. They found that the sensor sensitivity reached up to $0.03{\;\mathrm{kPa}}^{-1}$(0-15 kPa). Khan et al. [39] designed a highly conductive elastic sponge (GO-AgNF-PIsponge) composed of graphene oxide (GO), flower-shaped silver nanoparticles (AgNFs) and polyimide with a maximum sensitivity of $0.572{\;\mathrm{kPa}}^{-1}$ and a working range of 0–10 kPa. Furthermore, Ma et al. [40] immersed a polyurethane sponge in a mixed liquid of carbon nanotubes and graphene to obtain lightweight, compressible and highly sensitive piezoresistive sensors. The aforementioned research shows that adjusting the correlation between high-performance sensing materials and multi-state structures is an effective way to obtain high sensitive piezoresistive sensors.

In order to obtain piezoresistive sensors with high sensitivity and high stability, a simple manufacturing process, low cost, and a sensor that can meet different types of measurement capabilities, a novel fabrication method is presented in this study. In the proposed fabrication method, MWNTs were assembled into PDMS films with a pyramid microarray structure with a layer-by-layer self-assembly [41]. It should be indicated that layer-by-layer self-assembly (LBL) refers to a process of spontaneously forming a structurally stable molecular agglomerate or supramolecular structure between layers by using the layer-by-layer alternating bottom-up deposition method. This technique can be carried out with the help of weak interactions between molecules, such as electrostatic attraction [42]. The working principle of the flexible pressure sensor is as follows: the obtained PDMS film through the self-assembly process acts as the intermediate piezoresistive layer of the sensor. External pressure causes deformation of the pyramid microarray structure on the surface of the PDMS film. Then, the contact area between the electrode and the piezoresistive layer increases and a conductive path is formed between the MWNTs attached to the surface of the PDMS film and the electrode. Studies show that the larger the external pressure, the larger the contact area and the more the conductive paths. The resistance of the sensor varies as the pressure changes so that a pressure sensor capable of detecting external tactile is obtained.

In order to verify the rationality of the experiment, six types of samples with and without the pyramid microarray structure, with 5, 10, and 15 self-assembled layers were prepared for the test. Moreover, in this study, the flexible sensor was studied as a strain sensor. Touch is the key to the function of human hands, so touch sensors must be integrated into robot hands to improve them [43]. Finally, in order to prove the applicable potential of the sensor, the prepared flexible pressure sensor was installed on the five fingers of a glove, and the feedback of the tactile force was investigated by grasping objects with different weights. Meanwhile, the strain sensors were also installed on the back joints of the middle finger to provide feedback on the degree of joint bending.

The specific fabrication method and the process of the flexible pressure sensor is discussed in Section 2. Furthermore, the third section presents the various sensing characteristics of the flexible pressure sensor and analyzes the testing results. Finally, the summary and the conclusion of the present study is presented in Section 4.

## 2. Design and Fabrication

### 2.1. Experimental Materials

In this experiment, the high-purity MWNTs were supplied by Nanjing Xanano Materials Tech Co., Ltd. (Nanjing, Jiangsu Province, China). It should be noted that the purity, leaf diameter, leaf length and the carboxyl group content of the MWNTs were 95%, 10–20 nm, 10–30 um and 2.00 wt%, respectively. In order to disperse MWNTs in distilled water, the dispersant of the carbon nanotubes aqueous solutions provided by the company were used. The mass fraction of the MWNTs solution, which should be prepared, was 2.00 wt%. Then, the preset solution of MWNTs was prepared and ultrasonicated for 2 h at low temperature. Moreover, in order to better disperse MWNTs in water, agitation was performed each half hour during the ultrasonic process. After the ultrasonication, the preset solution was statically precipitated for 12 h. The precipitate in the bottom layer was poured out, and the upper layer solution was kept for further use. Moreover, the PDDA solution of 3.00 wt% (supplied by Wuxi Lansen Chemical Products Co., Ltd., Wuxi, Jiangsu Province, China) and the PSS solution of 3.00 wt% (supplied by Shanghai Yuanye Biotechnology Co., Ltd., Shanghai, China) were prepared, respectively. Then 0.5 mL of the NaCl solution was added to the aforementioned solutions to enhance the strength of the ions, increase the adsorption capacity of the ions, thereby improving the efficiency of the self-assembly process.

Asilicon wafer was prepared with a crystal orientation of <100>, thickness of 400 μm and an area of 4 inches. Moreover, a silicon dioxide (SiO_2_) layer with a thickness of 2 μm was utilized as the substrate for etching the microarray structure. According to the requirements of the silicon wafer etching, a wet-etching solution (BOE) of silicon dioxide (NH4F: HF = 5:1, *v*/*v*) and anisotropic wet etching KOH solution of the silicon layer with a concentration of 35% was prepared. Finally, the PDMS (Sylgard 184; Dow Corning Corp., Gales Ferry, Connecticut, USA) with a pre-formed mixture was prepared in accordance with the mass ratio of the curing agent to the substrate of 1:15. Then, the mixture was stirred and vacuum-treated for 10 min for further use.

### 2.2. Fabrication Process of the PDMS Substrate with the Pyramid Microarray Structure

**Figure 1:**
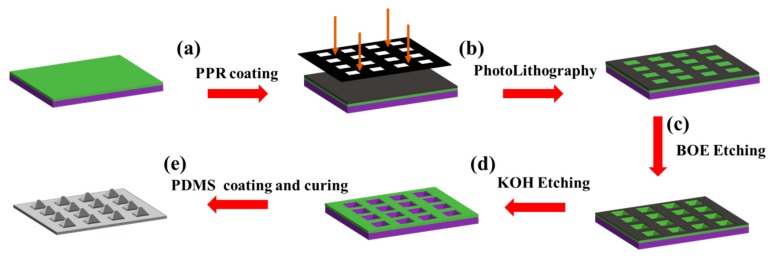
Fabrication process of the PDMS substrate with a pyramid microarray structure: (**a**) photoresist spin-coated on silicon wafer; (**b**) Silicon development; **(c)** wet etching SiO_2_ layer; (**d**) anisotropic etching of Si layer by KOH solution; (**e**) spin-coated stripping PDMS film.

Figure 1 shows the fabrication process flow of a PDMS film substrate with a pyramid microarray structure. According to the process flow, a layer of photoresist (PPR) is evenly spin-coated on the prepared silicon wafer initially. Figure 1a shows that the film mask was designed in advance according to the required microarray structure. Moreover, Figure 1b shows that the patterns on the designed film mask were transferred to the photoresist-coated silicon wafer by the development exposure. Then, the silicon wafer was placed in a wet etching solution (BOE) and the photoresist on the surface of the silicon wafer was used as a protective layer. Figure 1c shows that the exposed SiO_2_ layer was etched for 15 min, while the external water bath temperature was maintained at 45 °C. Then, the photoresist (PPR) was removed with an acetone solution, and the SiO_2_, which was not etched in the previous step was used as a protective layer. Figure 1d illustrates that the silicon wafer was immersed in the 30% KOH solution for 3 h to perform silicon layer (Si) etching, while the external water bath temperature is maintained at 80 °C. After etching, the inverted pyramid structure grooves were obtained on the silicon wafer. Then, the prepared PDMS preset solution was spin-coated onto the etched silicon wafer mold and solidified in the oven at 80 °C for 3 h. Finally, the PDMS film with the pyramid microarray structure was obtained after being stripped from the mold, which is shown in Figure 1e.

### 2.3. Design Process of the Multi-Walled Carbon Nanotubes Layer-by-Layer Self-Assembly

**Figure 2:**
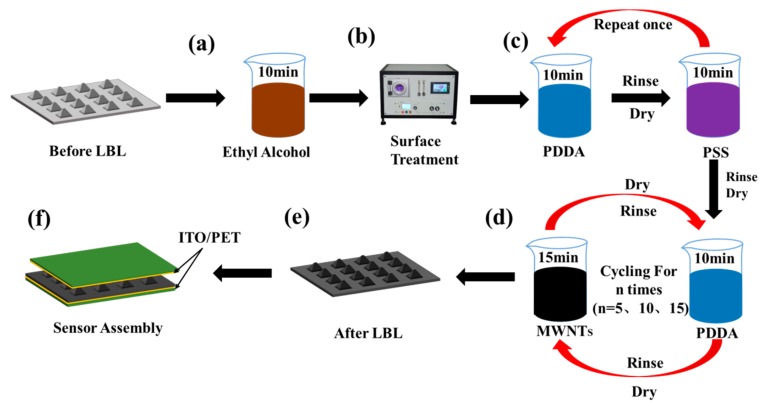
Fabrication process of the layer-by-layer self-assembly: (**a**) rinse using ethanol solution for 10 min; (**b**) treatment by oxygen plasma cleaner for 100 s; (**c**) pre-treatment using PDDA and PSS solutions; (**d**) MWNTs 5-10-15 layers self-assembly process; (**e**) PDMS file samples after layer-by-layer self-assembly; (**f**) the sensor consisting of two upper and lower ITO/PET electrode films and a layer-by-layer self-assembled PDMS film in the middle.

**Figure 3:**
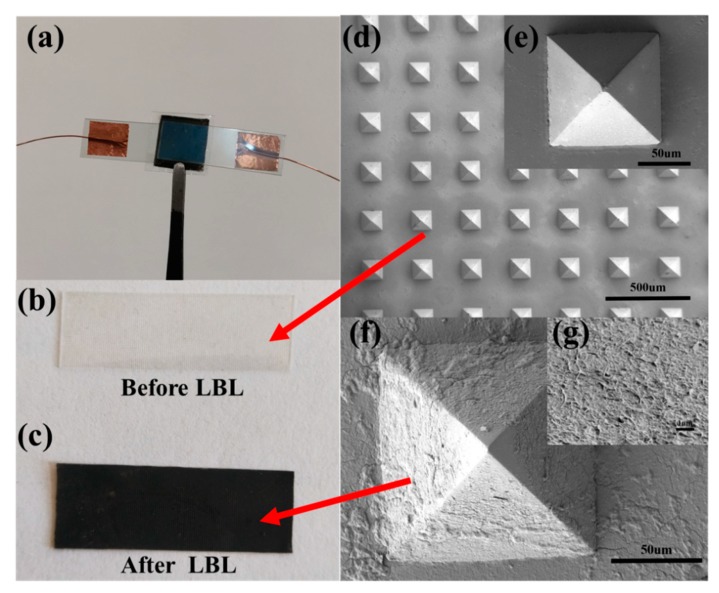
Physical images of PDMS films before and after the self-assembly process and SEM images: (**a**) photo of the actual sensor; (**b**) physical image of the PDMS film before self-assembly; (**c**) physical image of the PDMS film after self-assembly; (**d**) SEM image of the PDMS film before self-assembly; (**e**) SEM image of a single pyramid structure unit before self-assembly; (**f**) SEM image of a single pyramid structure unit after self-assembly; (**g**) SEM image of MWNTs meshed structure on the pyramid surface after self-assembly.

The MWNTs layer-by-layer self-assembly process was a key step in the fabrication of the intermediate piezoresistive layer of the pressure sensor. Figure 2 shows the specific fabrication process. Figure 2a shows that the PDMS film was first rinsed in ethanol for 10 min to remove surface stains and dust. It was then rinsed in the oxygen plasma cleaner for 100 s as shown in Figure 2b with the purpose of improving the surface activity of the PDMS, making it hydrophilic and increasing the ion adsorption. The pretreated PDMS film was immersed in the PDDA solution for 10 min, rinsed with ionized water and blown dry with nitrogen. Then, the pretreated PDMS film was immersed in the PSS solution for 10 min, rinsed with deionized water, and blown dry with nitrogen. Figure 2c illustrates that the aforementioned steps were repeated to insert the PDMS film into a preset body that was processed by PDDA and PSS solutions to enhance the adsorption of the charge. Then, the processed PDMS file preset body was alternately immersed in the PDDA and MWNTs solution for 10 min and 15 min, respectively and this step was repeated once. Figure 2d shows that after the processed PDMS file preset body was immersed in each solution, it was rinsed with deionized water and blown dry with nitrogen. After these steps, the PDMS films obtaining a non-pyramid microarray structure with a correlation of (PDDA/PSS)2 (PDDA/MWNTs)n and pyramid microarray structure was obtained. It should be indicated that n represents the number of the self-assembly (in this experiment, n = 5, 10, and 15). Figure 2e shows that the PDMS film after layers of self-assembly. Figure 2f shows that the self-assembled PDMS film samples were closely attached to the middle of two flexible polyethylene terephthalate (PET) films coated with conductive indium tin oxide (ITO) and wrapped with PDMS film outside to form a sandwich structure. Figure 3a shows the real picture of the encapsulated sensor.

Figure 3b,c illustrate the change of the PDMS film before and after self-assembly. An electron microscopy (SEM) scanning of the PDMS film prior to self-assembly and after self-assembly of 10 times was performed to verify the effectiveness of the self-assembly experiment. Figure 3d–g show the obtained results. Figure 3d shows that there were pyramidal cone structures with a regular arrangement and were the same size on the surface of the PDMS film without self-assembly. Moreover, Figure 3e shows that the surface of a single pyramid structure unit was smooth. Figure 3f shows that after the completion of the self-assembly, the surface of the single pyramid structure unit became rough due to the deposition of the dense and random MWNTs on the surface of the film. By further magnifying the carbon nanotube layer on the surface of the pyramid, it was observed that a large number of multi-walled carbon nanotubes were crisscrossed and interconnected to form a network structure (Figure 3g), which indicated that the experiment achieved the desired effect that a layer of MWNTs had been deposited on the surface of PDMS through the self-assembly process.

## 3. Results and Discussion

**Figure 4:**
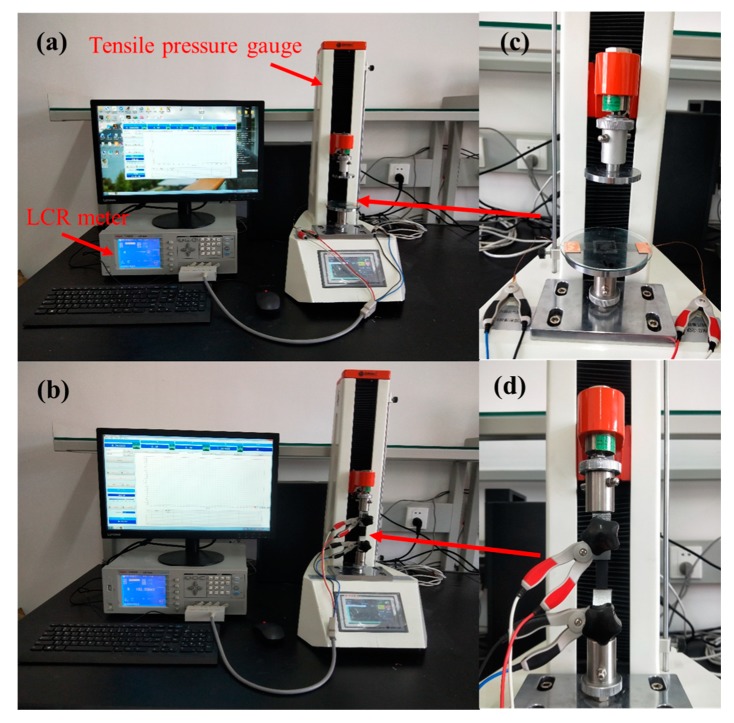
Experimental setup for sensor testing: (**a**) pressure testing process diagram; (**b**)strain force testing process diagram; (**c**) a partially enlarged view of (**a**); (**d**) a partially enlarged view of (**b**).

Figure 4 shows the experimental setup for testing a flexible pressure sensor. It indicates that the clamp of the pressure gauge can be changed to adjust the applied force in both directions. It is intended to squeeze and stretch the sensor to evaluate the tactile-force and strain-force performance of the sensor. The tensile pressure gauge was ZQ-990A (supplied by Dongguan Zhiqu Precision Instrument Co., Ltd., Dongguan, Guangzhou Province, China), where the measuring range varied from 0 N to 50 N with a force resolution of 0.01 N. Moreover, the test instrument of the resistance parameter was a desktop LCR meter (TH2826; Changzhou Tonghui Electronics Co., Ltd., Changzhou, Jiangsu Province, China). It was observed that the assembled pressure sensor was installed on the glass piece so that the electrodes were pulled out by a conductive tape with thin copper wires. During the test, the applied force varied from 0 to 4 kPa. Figure 4a indicates that in order to distribute the applied pressure evenly across the sensor, a microscope cover slip should be placed on the sensor surface. Figure 4b shows that for conducting the strain-stretching test, both ends of the PDMS film were fixed by clamps and the stretching displacement was set to 0–16.25 mm. In other words, the strain range was set to 0–65% and the stretching rate of the test instrument was 100 mm/min. Figure 4c,d show a partially enlarged view of the sensor tactile test and strain test.

### 3.1. Pressure Characteristics of Sensors

In the experiment, six samples were used to perform the sensitivity testing. Three samples of 5-10-15 times self-assembly had no pyramid microarray structure (here called as the L-N5, L-N10 and the L-N15, respectively), while the other three samples with 5, 10, and 15 times self-assembly had a pyramid microarray structure (hereafter called as the L-5, L-10 and the L-15, respectively). The sensitivity of a resistive sensor was defined as:

(1) $$S=\delta (\Delta R/R_{0})/\delta P$$

where $\Delta R=R-R_{0}$, P is the applied pressure, R is the resistance when pressure is applied to the device, and $R_{0}$ is the initial resistance without applied pressure.

**Figure 5:**
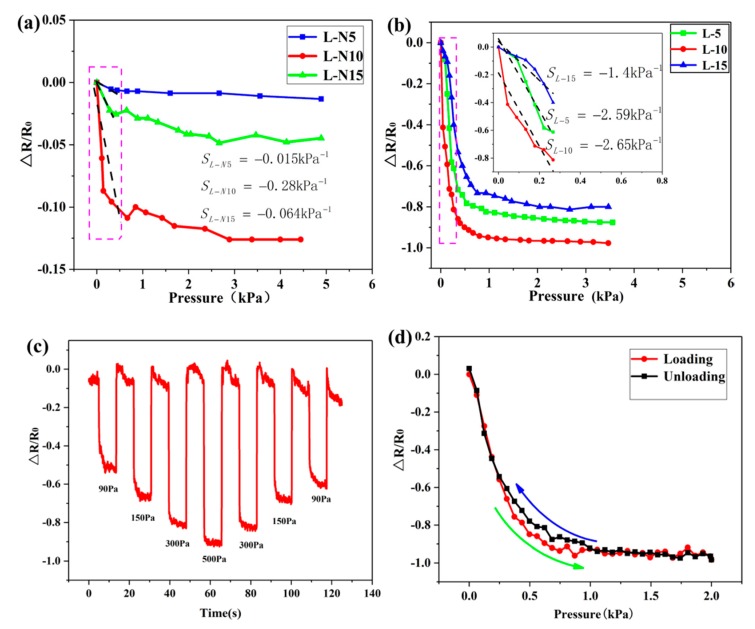

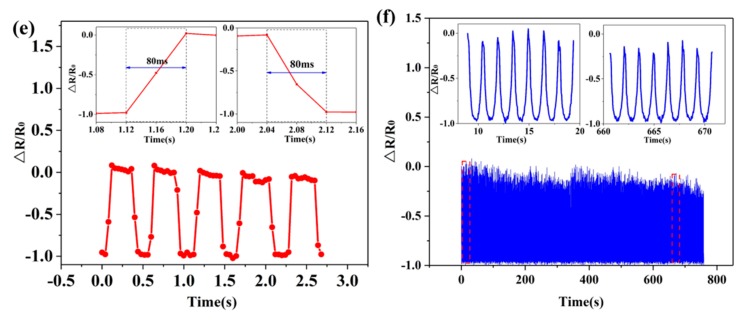
Performance tests of the L-10 flexible pressure sensors: (**a**) pressure response curve of the pressure sensor without a microarray structure; (**b**) pressure response curve of the sensor with a microarray structure; (**c**) response curve of the L-10 sensor dynamic loading/unloading different pressure; (**d**) relative resistance variation curve of the L-10 pressure sensor for two consecutive loading and unloading cycles; (**e**) step response time of L-10 pressure sensor; (**f**) resistance variation curves of L-10 pressure sensor under continuous 500 times repeated loading conditions.

A pressure of 0 to 4 kPa was applied to the sensor surface to measure the resistance at different pressures. Figure 5a,b show the correlation between the resistance and the pressure. Figure 5a indicates that sensors without pyramid microarray structure produce an extremely low response in the low-pressure range. In the range of 0 to 450 Pa of the sensor without the microarray structure, it was observed that the sensitivity increased as the number of self-assembled layers increased so that the maximum sensitivity approached $S_{L-N10}=-0.28{\;\mathrm{kPa}}^{-1}$ (<450 Pa) when the number of self-assembled layers was 10. Figure 5b presents the variation of the sensor sensitivity with the pyramid microarray structure. It indicates that the resistance of three sensors decreased as the pressure increased. This phenomenon was interpreted from a mechanical point of view. When the external pressure was applied to the sensor surface, the pyramid structure was deformed accordingly, and the contact area between the carbon nanotubes and the electrodes on the pyramid microarray structure increased. Then, the number of conductive paths between them increased, which led to a decrease in device resistance. The experimental results show that the pressure response curve of the sensor with the microarray structure could be divided into two segments. When the pressure varied from 0 to 300 Pa, the variation in the sensitivity was remarkable. This may be attributed to the deformation of the elastic cone PDMS, which abruptly increased the contact area between the PDMS film and the ITO/PET electrode so that the surface MWNTs and electrodes rapidly generated a large number of conductive paths with low pressures. Finally, this deformation resulted in a sharp drop in the resistance. On the other hand, when the pressure exceeded 300 Pa, the resistance changed slowly. This may be attributed to the saturation of the pyramid deformation on the PDMS film so that any additional pressure can only cause tiny changes in the contact area so that the number of conductive paths is not affected significantly.

Experiments show that the initial resistances of L-5, L-10, and L-15 were 1200 KΩ, 750 KΩ, and 75 KΩ, respectively. It was observed that the initial resistance of the sensor decreased as the number of MWNTs layers increased. This originates from the fact that more deposited MWNTs on the surface of the PDMS film, creates more conductive paths so that the conductivity increases, and the initial resistance reduces. The illustration in Figure 5b shows the sensitivity fitting of the sensor with the microarray structure in the range of 0 to 300 Pa. Experiments show that the sensitivity of the L-10 sensor was the highest and approached $S_{L-10}=-2.65{\;\mathrm{kPa}}^{-1}$. Moreover, it was followed by the L-5 sensor with a sensitivity of $S_{L-5}=-2.59{\;\mathrm{kPa}}^{-1}$, while the L-15 sensor had the lowest sensitivity, which was only $S_{L-15}=-1.4{\;\mathrm{kPa}}^{-1}$. It was found that the sensor sensitivity did not increase as the MWNTs increased.

This phenomenon can be explained as follows. The content of multi-walled MWNTs on the surface of L-5 was less than that of L-10. At the same pressure, when MWNTs on the L-10 surface were in contact with the ITO conductive electrodes, the corresponding conductive paths were more than that generated by the L-5 sensor. Therefore, the sensitivity was high and the resistance variation of the L-10 sensor was larger than that of the L-5 sensor.

Moreover, the initial resistance of the L-15 sensor was the smallest, because a large number of MWNTs were deposited on the surface of the PDMS film and the conductive paths generated between themselves were already abundant. However, when the pressure was applied to the L-15 sensor, the ratio of the resistance originating from the generated conductive paths, when the surface MWNTs were in contact with the ITO conductive electrodes, to the initial resistance was much smaller than that of the L-10 sensor. Therefore, the L-10 sensor was the most sensitive layer of the three different MWNTs layers. The most sensitive L-10 sensor with the microarray structure was compared with the most sensitive L-N10 sensor without the microarray structure in the low-pressure range. It was found that the sensitivity of the sensor with the microarray structure was 9.46 times more than the sensor without the microarray structure, which meant that the design with the microarray structure improved the sensitivity performance of the sensor. Furthermore, since the sensitivity of L-10 was the highest, in the following tests the flexible pressure sensor with 10 layers of self-assembled MWNTs was used as the test sample.

In addition to high sensitivity, sensors must have other properties such as a fast response, good hysteresis characteristics, repeatability and the ability to detect multiple types of pressure. Figure 5c displays the dynamic characteristics of the loading and unloading pressure of the sensor. Moreover, loading and unloading tests are carried out at different pressures, including 90 Pa, 150 Pa, 300 Pa and 500 Pa. The obtained results show that the sensor had good regularity and stability. It should be indicated that the hysteresis characteristics of the sensor can cause deviations in the pressure readings of the dynamically loaded and unloaded sensors at the same pressure, which affects the reliability of the sensor. Figure 5d shows that in order to verify the reliability of the sensor’s dynamic loading process, the piezoresistive response curves under the same pressure of loading and unloading were measured in the continuous cycle of 0 to 2 kPa. Moreover, changes in the resistance hysteresis characteristics were analyzed based on test results. It was observed that the resistance of the sensor did not change significantly during the pressure loading and unloading, which indicated that the pressure sensor had reasonable stability and reliability during the dynamic loading/unloading process.

Figure 5e shows the response curve and relaxation time of the sensor when the pressure is 1 kPa step signal. It was observed that the response and relaxation time of the sensor were both 80 ms, indicating that the pressure sensor had a faster response capability in practical applications. Moreover, the durability of the sensor under pressure was tested by repeatedly loading/unloading a pressure of 0.2 N and continuous cycling of 500 times. Figure 5f shows that the sensor maintained reasonable stability during the test without significant performance degradation. Moreover, it was observed that the resistance change curves of the repeated tests in two different periods were very close, and no attenuation occurred. This indicates that the sensor maintained excellent durability under severe repetitive pressure conditions.

### 3.2. Strain Characteristics of the Sensors

**Figure 6:**
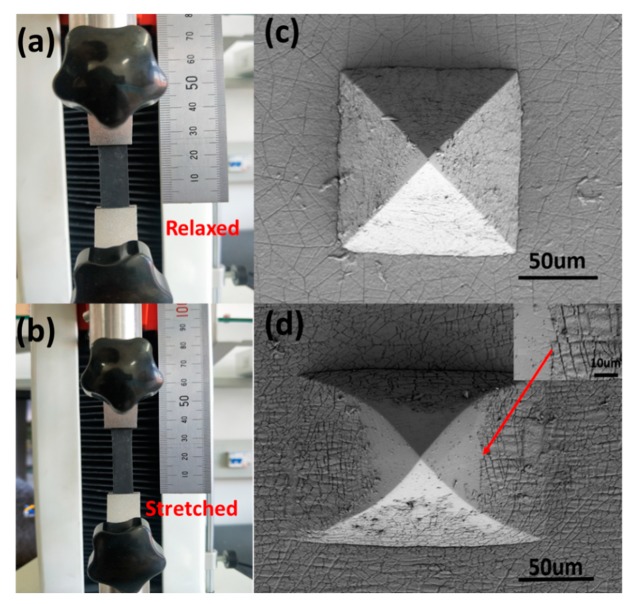
Test physical images of sample L-10 as a flexible strain sensor after being relaxed and stretched and surface SEM scanning images: (**a**) test physical image of strain sensor in a relaxed state; (**b**) test physical image of strain sensor being stretched 40%; (**c**) surface SEM scanning image of strain sensor in a relaxed state; (**d**) surface SEM scanning image of a strain sensor after being stretched.

During the experiment, it was found that when the PDMS film was subjected to the tensile force, its resistance produced a corresponding stress change. Therefore, the strain resistance change caused by stretching the L-10 sample with the pyramid microarray structure and 10 layers of self-assembled layers was analyzed. Figure 6a,b show the obtained results. Figure 6a illustrates the state of the sensor before it was subjected to the tensile force, while Figure 6b shows the state of the sensor when tension was applied with 40% of its own deformation. After stretching, it was observed that the film was not broken, indicating that the film has strong flexibility and meets the requirements for practical applications.

**Figure 7:**
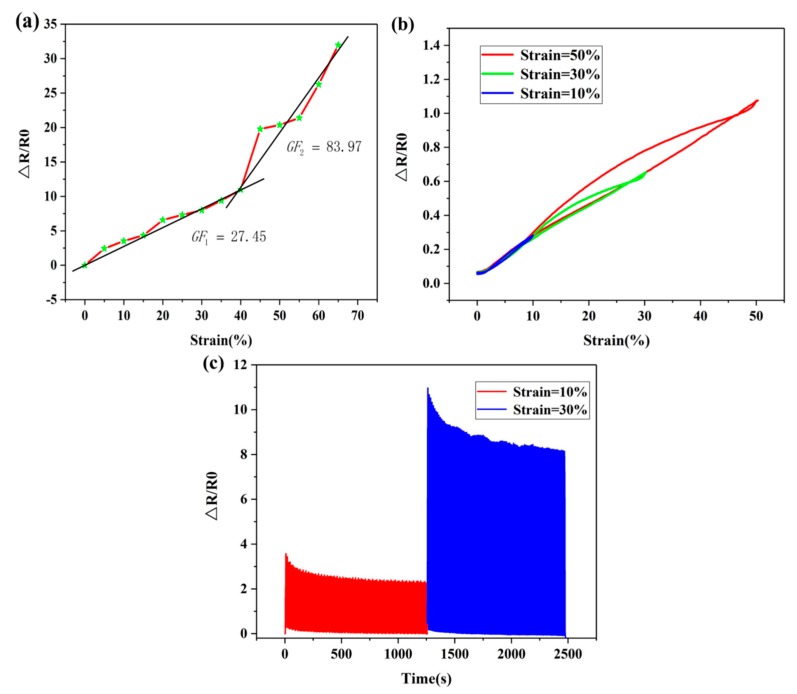
Characteristics of the L-10 sensor sample as a strain sensor: (**a**) the corresponding curve of variations of resistance and tensile deformation of the strain sensor from 0% to 65%; (**b**) hysteresis characteristics of the strain sensor under tensile-shrink strain with tensile deformation of 10%, 30%, and 50%; (**c**) the dynamic response of the strain sensor repeating the stretching cycle 100 times with tensile deformation of 10% and 30%.

Moreover, Figure 7a shows the relationship between the variation amount in the resistance ($\Delta R/R_{0}$) and the change amount in the tensile length when the sensor is stretched. The equation of gauge factor (GF) of the sensor was as follows:

(2) $$\mathrm{GF}=(\Delta R/R_{0})/\varepsilon$$

where ΔR is the resistance change with straining, $R_{0}$ is the resistance before straining and ε is the relative change of the stretching length of the sensor.

Figure 7a shows that during the stretching process, the resistance of the film continuously increased. When the tensile strain was between 0% and 40%, GF was 27.45. However, when the tensile strain exceeded 40%, the rate of the variation in the resistance rapidly increased with GF of 83.97. Moreover, the surface morphology of the film samples without stress and with strain of 40% was scanned by the SEM. Figure 6c shows that the carbon nanotubes on the surface of the film were closely linked together and no fracture occurred in a relaxed state without being subjected to tensile force. However, Figure 6d shows that under the tensile force, the PDMS pyramid microarray structure of the sensor was laterally deformed, and the carbon nanotube layer on the surface of the film was broken and gaps appeared, which caused the decrease in the conductive paths between the sensor carbon nanotubes and the increase in the resistance of the sensor. Moreover, when the length deformation of the sensor was more than 40%, more fractures occurred randomly on the film, the gaps gradually increased, the conductive paths continuously decreased and the variation amount in resistance of the sensor rapidly increased. In order to demonstrate the tensile strain hysteresis characteristics of the sensor, the 10%, 30% and 50% tensile-shrink strain periods of the tensile deformation were designed. Figure 7b shows the variation amount in the resistance of the sensor and the strain characteristic curves. The obtained results demonstrate that the sensor had no obvious hysteresis characteristics at the tensile deformation of 10%, while the hysteresis characteristic was significant at a tensile deformation of 30%. Compared with tensile deformation of 10% and 30%, a more obvious hysteresis appears at the tensile deformation of 50%. A reasonable explanation is that under low tensile force in the sensor, the damaged conductive paths can recover quickly within a small stress range. As the tensile action increases, the fracture of the carbon nanotube on the sensor surface increases continuously and therefore gaps gradually increase. Under the action of high strain, the blocked conductive paths take more time to return to the original state. Therefore, the hysteresis becomes obvious. The optional stretching range of the sensor was selected to be within 0–30%. In order to examine the dynamic tensile stability of the sensor, the sensor was fixed on the test instrument. Moreover, the stretching range was set to 0–10% and 0–30%. Stretchingand releasing tests were performed 100 times in a given range, respectively. Figure 7c indicates that the sensor had excellent repeatability and stability.

### 3.3. Feedback of the Tactile and Bending Degree

**Figure 8:**
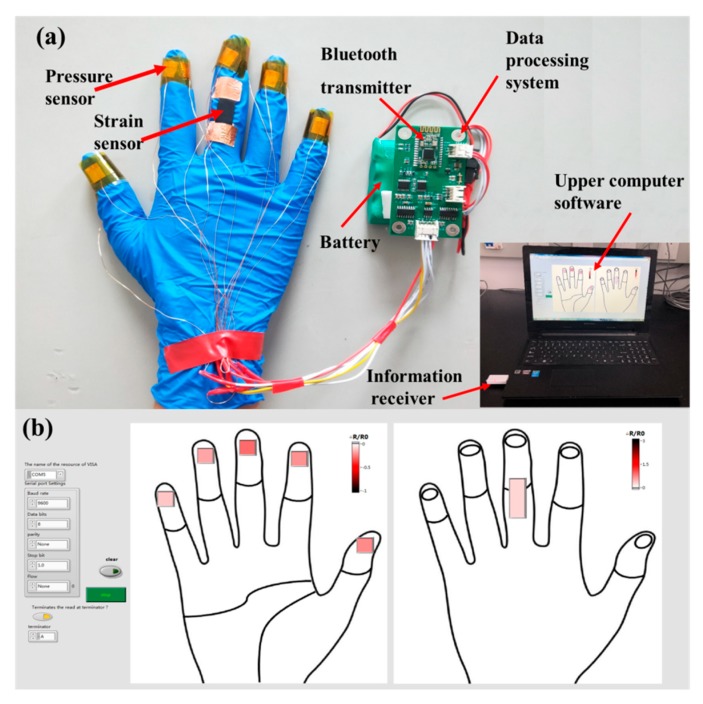
Sensor testing system: (**a**) physical image of the glove and sensor acquisition module; (**b**) upper computer interface of Labview.

As shown in Figure 8a, we used a combination of a hardware circuit and software system to verify the effectiveness of the sensor. The sensor was installed on the five fingers of a glove as a pressure sensor to achieve feedback on the tactile force when grabbing objects with different weights. Moreover, the sensor was installed on the joint of the back of the middle finger as a strain sensor to achieve feedback on the joint bending degree when grabbing objects with different volumes. At the same time, the data acquisition and processing circuit of the lower computer was designed. The circuit was powered by mobile power (the battery voltage was 4.8 V). The upper computer display interface was designed by using Labview software. Using the Bluetooth wireless transmission technology, the transmission module sends the data processed by the lower computer to the receiving module on the PC port, so that the data was displayed in real time on the upper computer interface. Figure 8b shows the host computer interface, which visually demonstrated the relationship between the amount of change in resistance and the intensity of the touch and the degree of bending using the change in color intensity.

**Figure 9:**
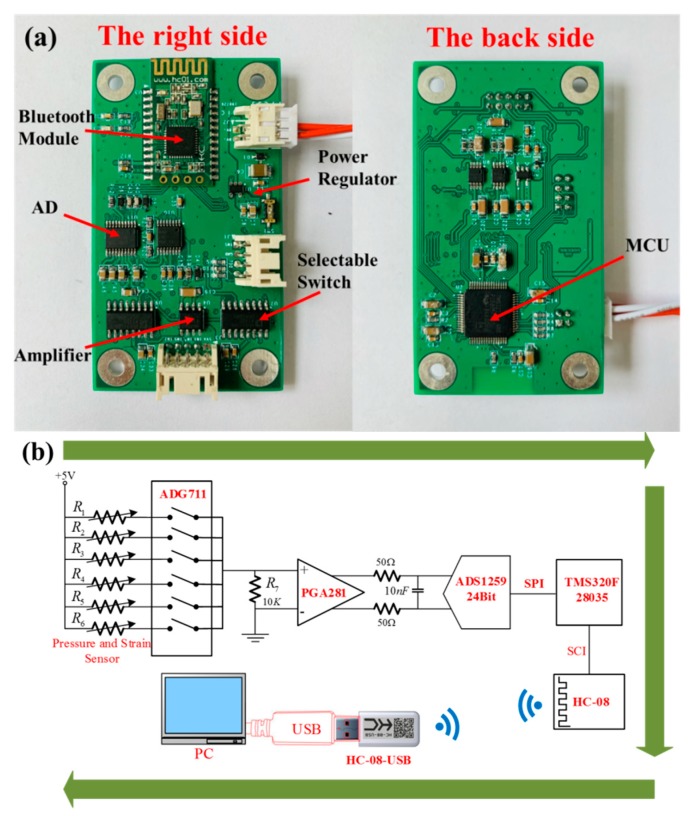
Hardware circuit and design block diagram: (**a**) the hardware circuit on the front and back of the data acquisition board; (**b**) block diagram of signal acquisition and processing design.

Figure 9a shows the hardware circuit on the front and back of the data acquisition board of the lower computer. The MCU was TMS20F28035, 24-bit analog-to-digital converter was ADS1259, programmable gain instrumentation amplifier was PGA281, selectable switches wereADG711, Bluetooth module was HC-08. Figure 9b shows a basic block diagram of data acquisition, processing, and display. $R_{1}–R_{5}$ represents the five tactile sensors on the finger, $R_{6}$ represents the strain sensor on the middle finger, and $R_{7}$ represents the sampling resistance with a resistance of 10 KΩ and an accuracy of 0.05%. The process of data acquisition was as follows: When we take gloves and grab the beaker and ball, the ADG711 turns on the switch of $R_{1}–R_{6}$ in turn, and the current flows through the sensor and $R_{7}$ to form a conductive loop, and the ADS1259 collects the weak voltage at both ends of $R_{7}$ and performs differential amplification and digitations. After being processed by MCU, the digital value is transmitted to the upper computer through Bluetooth. We obtained the pressure change value of $(R-R_{0})/R_{0}$ and expressed the pressure change with the color depth on the upper computer.

**Figure 10:**
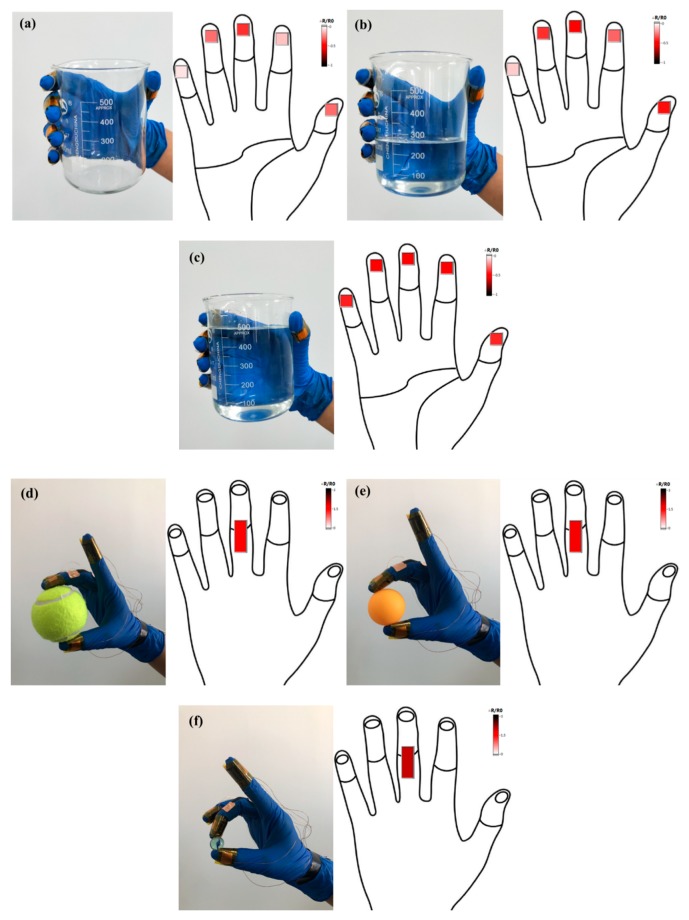
Changes in the finger tactile force and bending degree of finger joints of the glove when grabbing objects of different weights and shapes: (**a**–**c**) The resistance change corresponding to the finger tactile force of the glove when grabbing an empty beaker, a beaker with 250 mL water and a beaker with 500 mL of water, respectively; (**d**–**f**) The resistance change corresponding to the joint bending degree of the glove when grabbing a tennis ball, a ping pong ball and a glass marble, respectively.

As shown in Figure 10a–c, the feedback about the tactile force was studied by using a 500 mL beaker as a carrier in three statuses, including empty, containing 250 mL water and 500 mL water. Through the display interface, we could see that the glove grabbed the beaker of different weights, and the touch sensor of each finger had different color changes, indicating that the value of resistance changed differently. It was observed that the intensity graph of the tactile force was the darkest when the beaker was filled with 500 mL water, while the variation in the resistance of the sensor was the highest, which indicated that the gripping force of the glove was the highest at this time. As shown in Figure 10d–f, in the feedback experiment of the joint bending degree, a tennis ball, a ping pong ball and a glass marble were used as grabbing objects. Through the display interface, we could see that as the ball became smaller and smaller in diameter, the color feedback of the sensor behind the middle finger got darker and darker. When grasping the glass ball with the smallest diameter, the strength diagram with the greatest degree of bending was the darkest color and the largest change in resistance, which indicated that the degree of bending of the finger joint was the greatest. The feedback results of tactile force and joint bending indicated that the results were consistent with human grasping characteristics. The experimental results showed that the designed flexible sensor could not only measure the perceptual change of the tactile size, but it could also indirectly reflect the bending degree of the joint, which provides a new method for robot precise control and intelligent prosthetic hand and human interaction, which has broad application prospects.

## 4. Conclusions

In the present study a novel method for constructing a highly sensitive flexible pressure sensor was proposed. This method combined MWNTs with PDMS films and pyramidal microarray structure arrays. The experimental results show that the PDMS film with 10 self-assembled layers serves as a piezoresistive layer. Then, a high performance flexible piezoresistive sensor is obtained, which obtains high sensitivity to $-2.65{\;\mathrm{kPa}}^{-1}$(<300 Pa), fast response speed (80 ms), reasonable flexibility and reliable stability. Moreover, compared with the structure without the microarray structure, the microarray structure improves the performance of the sensor and significantly enhances the sensitivity of the sensor. Furthermore, it was observed that the sensitivity of the sensor depended on the number of layers of the MWNTs self-assembly. Therefore, sensors with different sensitivity requirements can be obtained by adjusting the number of layers, which makes it possible to design different sensors for different applications. Finally, it was successfully verified that the designed sensor not only obtained excellent performance in tactile detection, but could also be used as a strain sensor to achieve better detection of the bending degree, which provides a new design concept for the application of flexible sensors in intelligent prosthetic hands, special operating robots, service robots and human-computer interaction.
